# Comparative analysis of transcriptional profiling of CD3+, CD4+ and CD8+ T cells identifies novel immune response players in T-Cell activation

**DOI:** 10.1186/1471-2164-9-225

**Published:** 2008-05-16

**Authors:** Min Wang, Dirk Windgassen, Eleftherios T Papoutsakis

**Affiliations:** 1 Interdepartmental Biological Sciences Program, Northwestern University, Evanston, IL, USA; 2 Immunotherapy Development, Dendreon Corporation, Seattle, WA, USA; 3 Department of Chemical and Biological Engineering, Northwestern University, Evanston, IL, USA; 4 Department of Chemical Engineering and the Delaware Biotechnology Institute, University of Delaware, Newark, DE, USA

## Abstract

**Background:**

T-cell activation is an essential step of the immune response and relies on the tightly controlled orchestration of hundreds of genes/proteins, yet the cellular and molecular events underlying this complex process are not fully understood, especially at the genome-scale. Significantly, a comparative genome-scale transcriptional analysis of two T-cell subsets (CD4+ and CD8+) against each other and against the naturally mixed population (CD3+ cells) remains unexplored.

**Results:**

Comparison of the microarray-based gene expression patterns between CD3+ T cells, and the CD4+ and CD8+ subsets revealed largely conserved, but not identical, transcriptional patterns. We employed a Gene-Ontology-driven transcriptional analysis coupled with protein abundance assays in order to identify novel T-cell activation genes and cell-type-specific genes associated with the immune response. We identified potential genes involved in the communication between the two subsets (including IL23A, NR4A2, CD83, PSMB2, -8, MIF, IFI16, TNFAIP1, POU2AF1, and OTUB1) and would-be effector-function-specific genes (XCL2, SLAMF7, TNFSF4, -5, -9, CSF3, CD48 and CD244). Chemokines induced during T-cell activation, but not previously identified in T cells, include CCL20, CXCL9, -10, -11 (in all three populations), and XCL2 (preferentially in CD8+ T cells). Increased expression of other unexpected cytokines (GPI, OSM and MIF) suggests their involvement in T-cell activation with their functions yet to be examined. Differential expression of many receptors, not previously reported in the context of T-cell activation, including CCR5, CCR7, IL1R2, IL1RAP, IL6R, TNFRSF25 and TNFRSF1A, suggests their role in this immune process. Several receptors involved in TCR activation (CD3D, CD3G, TRAT1, ITGAL, ITGB1, ITGB2, CD8A and B (CD8+ T-cell specific) along with LCK, ZAP70 and TYROBP were synchronously downregulated. Members of cell-surface receptors (HLA-Ds and KLRs), none previously identified in the context of T-cell activation, were also downregulated.

**Conclusion:**

This comparative genome-scale, transcriptional analysis of T-cell activation in the CD4+ and CD8+ subsets and the mixed CD3+ populations made possible the identification of many immune-response genes not previously identified in the context of T-cell activation. Significantly, it made possible to identify the temporal patterns of many previously known T-cell activation genes, and also identify genes implicated in effector functions of and communication between CD4+ and CD8+ T cells.

## Background

T cells are among the most versatile cells in the body and play a central role in adaptive immunity. T-cell maturation in thymus is a stepwise process, undergoing positive and negative selection to produce CD4+ and CD8+ T cells [1]. When mature, T lymphocytes leave the thymus and are considered naive cells until they encounter activating signals in peripheral lymphoid organs, thus become activated, start to proliferate, differentiate into effector cells (helper and cytotoxic) and gain the ability to enter inflammations sites [2]. Activation of the naive T cells in the peripheral immune system is the first step of the adaptive immune response.

Successful T-cell activation requires two major stimulatory signals to produce an effective immune response. First, the T-cell receptor complex (TCR) recognizes the cognate ligands presented by the major histocompatibility complex (MHC) on antigen-presenting cells (APCs) [3]. Second, a co-stimulation signal is presented to T cells through the engagement of a co-receptor such as CD28 [4]. In the absence of CD28 co-stimulation, TCR signalling alone results in anergy. T-cell activation, which begins with TCR activation with CD28 co-stimulation, triggers multiple signalling pathways and cellular events. Signalling downstream of TCR engagement has been widely studied [5-7]. Key events include activation of protein kinases such as LCK and ZAP70, intracellular Ca^2+ ^regulation, activation of MAP-kinase cascades, and activation and nuclear localization of crucial transcription factors including AP-1, NFAT, and NF-κB. However, our understanding of the activation process including subsequent proliferation and differentiation events is far from complete. A temporal genome-scale transcription profiling of T-cell activation process would provide a comprehensive understanding and insights into the molecular mechanisms underlying the process.

Gene expression analysis of T-cell activation at a single timepoint has been reported [8,9], and using a single donor sample, the gene expression patterns of T-cell activation with or without co-stimulation by anti-CD28 antibody were compared [10,11]. However, to the best of our knowledge, the genome-scale donor-independent temporal gene expression analysis of primary, human T-cell activation has not been reported, and this is the goal of this study. Significantly, a comparative analysis of the programs of two T-cell subsets (CD4+ and CD8+) against each other and against the natural CD3+ population remains unexplored, and would likely yield significant new information. Comparison of the transcriptional patterns among the three populations should lead to the identification of the common transcriptional events shared by CD4+ and CD8+ T cells, and of subset-specific genes and genes potentially involved in the communication between CD4+ and CD8+ T cells. Among the differentially expressed genes, we focused on 'immune response' genes based on Gene Ontology (GO) classification in order to provide new insights into the expression of chemokines and cytokines, the orchestrated regulation of receptors, the interactions between the two subsets, and the homeostasis of resting T cells. Such understanding would be helpful for enhancing, re-directing or modifying the activities of T cells under physiological and pathophysiological circumstances.

## Results

### Primary human T-cell activation is donor independent

**Figure 1:**
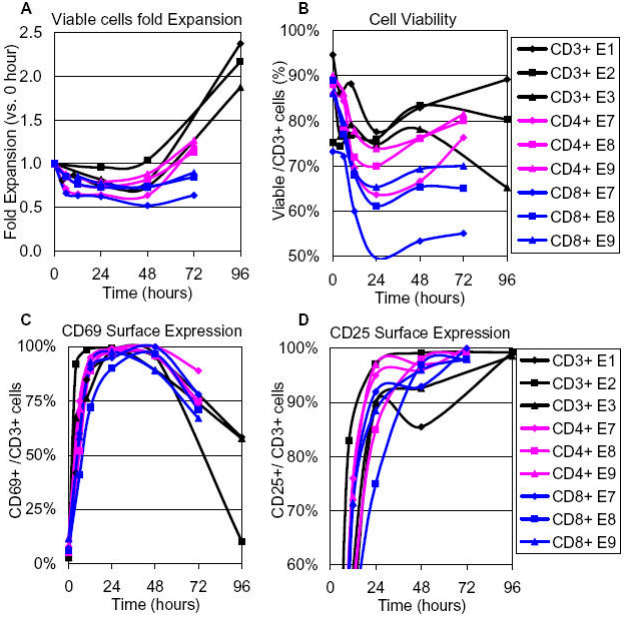
**Phenotypic analysis of T-cell ex vivo activation upon anti-CD3/anti-CD28 stimulation of three populations**. Three independent biological experiments were carried out for each population. CD3+, CD4+ and CD8+ T cells were negatively selected from PBMCs of healthy donors and activated with anti-CD3/anti-CD28 antibodies. **(A) **T-cell expansion as assessed by cell numbers; **(B) **The percentage of the viable T cells as determined by flow cytometry; **(C) **The percentage of the viable cells expressing CD69; **(D) **The percentage of the viable cells expressing CD25. Data from 6 independent experiments (CD3+ T-cell experiments, E1–E3, and CD4+ and CD8+ T-cell experiments, E7–E9) using cells from 6 different healthy donors are shown.

We aimed to capture important, donor-independent transcriptional events of T-cell activation. Three biological experiments, E1–E3, using CD3+ T cells, which contain both the CD4+ and CD8+ subsets, from three different healthy donors demonstrated similar phenotypic characteristics. T-cell proliferation, as measured by cell expansion, started at 48 hours after stimulation, and cell numbers doubled by 96 hours (Figure 1A). Cell viability remained around 80% throughout the 96 hours (Figure 1B). Surface expression of the early T-cell activation marker CD69 was rapidly upregulated within 10 hours, and then downregulated after 48 hours (Figure 1C). Expression of the other important surface marker CD25 (IL2RA) rapidly increased within 24 hours and stayed high (above 80%) from 24 hours to 96 hours (Figure 1D). We also examined the CD4+/CD8+ subset ratio but we found no significant changes during the 96 hours of the experiments (data not shown). CD4+ cells were ca. 60%, and CD8+ cells ca. 40% of the total T-cell population.

A separate set of experiments, E7–E9, was carried out using separately CD4+ and CD8+ T cells isolated from another three healthy donors. The time course analysis of this set of experiments was setup somewhat differently than in the CD3+ T-cell study (0, 6, 12, 24, 48 and 72 hours in the CD4+ and CD8+ subsets compared to 0, 4, 10, 48, and 96 hours in CD3+ T cells) in order to cover earlier timepoints. As we demonstrate below, the different time points in the two sets of experiments do not affect our ability to compare the data from the two studies, and in fact enhance and broaden the validity of the conclusions. Within each population, T cells exhibited overall similar phenotypic characteristics (Figure 1). T-cell proliferation as assessed by cell numbers did not start until 48 hours. Expansion reached about 1.2 fold in CD4+ T cells and about 0.8 fold in CD8+ T cells by 72 hours. Cell viability remained around 75% in CD4+ T cells vs. around 60% in CD8+ T cells; the lower viability of CD8+ T cells was likely caused by the absence of help from CD4+ T cells. Expression of the T-cell activation surface markers CD25 and CD69 in the two subsets was similar to that of CD3+ T cells.

**Figure 2:**
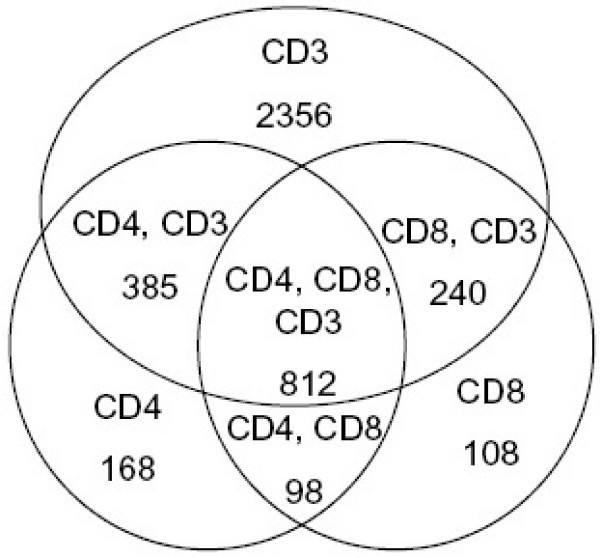
**Venn diagram comparison of the significant genes identified as differentially expressed in the three populations**. SAM analysis (false discovery rate of <1%) identified 3793 significant genes in CD3+ population, 1463 significant genes in the CD4+ population and 1258 significant genes in the CD8+ population.

Agilent microarrays that target 18,403 human genes were used to generate the transcriptional profile of activation for the CD3+ T-cell population, and the CD4+ and CD8+ T-cell subsets. Comparing samples across all time points, multi-class SAM (false discovery rate of <1%) identified 3793 genes with statistically significant expression changes in the CD3+ population, 1463 significant genes in the CD4+ population, and 1258 significant genes in the CD8+ population. Hierarchical clustering (see Additional files 1, 2, 3, 4, 5, 6) demonstrated that the transcriptional patterns of these significant genes among the replicate biological experiments within each population were highly reproducible. Thus, for simplicity and ease of presentation, gene expression data from the three biological experiments for each population were averaged for discussion and analysis below. In order to compare the transcriptional patterns of CD3+, CD4+ and CD8+ T cells, the significant genes from all experiments were combined to a total of 4167 unique, significant genes distributed among the 3 T-cell populations as shown in the Venn diagram of Figure 2. Far more significant genes were identified in the CD3+ T-cell activation experiments than in the subset experiments, possibly reflecting a larger repertoire of genes during activation in the natural, mixed population of CD3+ T cells, and thus the synergy and interplay of the two subsets (CD4+ and CD8+ T cells) in producing a more complex and multifaceted response. Nevertheless, a large number of the genes were shared by CD4+ and CD8+ T cells (910 out of 1463 and 1258 respectively), reflecting the common cellular events shared by CD4+ and CD8+ T cells during the activation process. Hierarchical clustering of these pooled significant genes (see Additional files 7 and 8) demonstrated that the 3 populations shared largely similar transcriptional profiles regardless of the difference of the sampled timepoints, which, in perspective, broadens the significance of identified genes.

Q-RT-PCR was used to validate select microarray results. Fifteen significant genes with different expression intensities were selected. As previously reported [12], in our laboratory, data from these Agilent microarrays correlated strongly with the Q-RT-PCR results, although Q-RT-PCR data generally show larger fold changes compared to microarray data (see Additional file 9). We thus conclude that the T-cell activation process under our experimental conditions is largely donor invariant, as assessed by both phenotypic data and transcriptional profiles.

### Regulation of ' immune response' genes in T-cell activation

**Figure 3:**
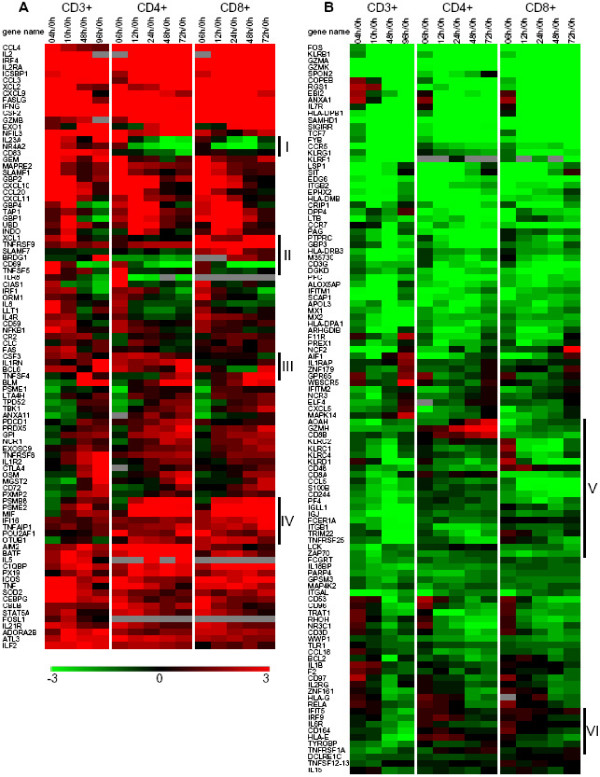
**Expression profiles of genes associated with the Gene Ontology term 'immune response'**. Genes that were differentially expressed temporally in T-cell activation of the three (CD3+, CD4+ and CD8+) populations were divided into two groups (**A **with mostly upregulated genes and **B **with mostly downregulated genes) according to their distinct expression patterns based on hierarchical clustering using the Euclidian distance metric. Color denotes degree of differential expression compared to 0 hour (saturated red = 3-fold up-regulation, saturated green = 3-fold down-regulation, black = unchanged, gray = no data available). Clusters (I-VI) of genes with different expression patterns among the three populations were noted on the side. Expression data shown are averages from three independent biological experiments for each T-cell population.

Ontological analysis using the MeV EASE module identified 203 genes associated with the term 'immune response' among the 4167 significant genes, consistent with the essential roles of T cells in the adaptive immune response. Hierarchical clustering revealed distinct expression patterns for these 203 genes and allowed us to divide them into two clusters: (A) Expression is mainly upregulated compared to resting T cells (0 hour) (Figure 3A); (B) Expression is mainly downregulated compared to resting T cells (0 hour) (Figure 3B).

Although we expected differences in gene expression patterns because of the different biology and functions of CD4+ and CD8+ T cells, the aforementioned 203 genes show, overall, similar expression patterns among the 3 populations but with several notable exceptions. Within the upregulated cluster A, notable differences among the three populations include: (1) genes in clusters I (IL23A, NR4A2, CD83) and IV (PSMB8, PSME2, MIF, IFI16, TNFAIP1, POU2AF1, and OTUB1), which shared similar gene expression patterns between CD4+ and CD8+ T cells, but different than those of CD3+ T cells; (2) genes in cluster II (XCL1, SLAMF7, BRDG1, CD69, TNFRSF9 and CD40LG (TNFSF5)) with different gene expression patterns among the 3 populations, and cluster III (CSF3, IL1RN, BCL6 and TNFSF4) with different gene expression patterns between the CD4+ and CD8+ populations. Within the downregulated cluster B, there were a few genes with different expression patterns in cluster V (AOAH, CD8A, -B, KLRC1, -2, -4, KLRD1, CD48, CCL5, S100B, CD244, PF4, IGLL1, IGJ, FCER1A, ITGB1, TRIM22, TNFRSF25, LCK and ZAP70), mainly between CD4+ and CD8+ T cells, and cluster VI (IFIT5, ISGF3G, IL6R, CD164, HLA-E, TYROBP and TNFRSF1A), mainly between CD3+ T cells and CD4+, CD8+ subsets.

**Figure 4:**
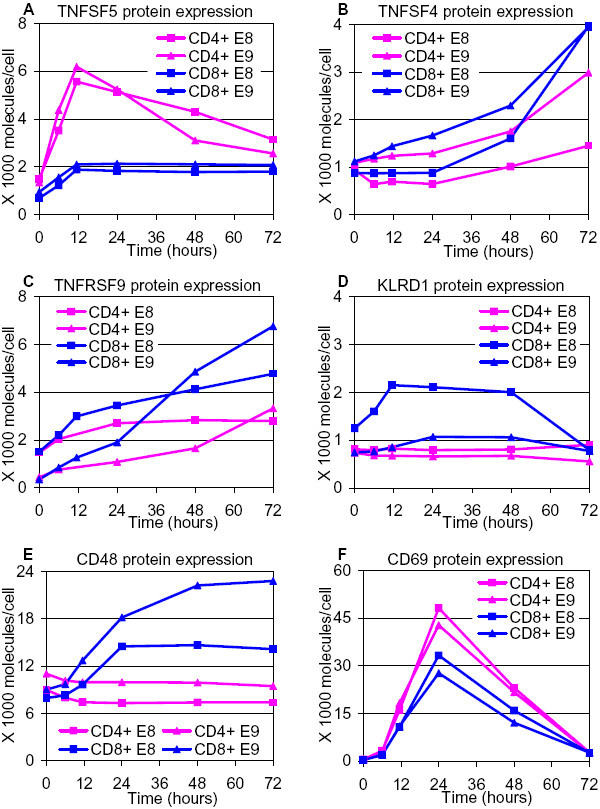
**Protein expression profiles supported the different transcription patterns between CD4+ and CD8+ subsets**. CD4+ T cells and CD8+ T cells were selected, stimulated (with anti-CD3/anti-CD28 antibodies), cultured separately and harvested at the indicated timepoints of culture. Flow cytometric assays were carried out for the selected genes with different transcription patterns between CD4+ and CD8+ subsets (**(A) **TNFSF5, **(B) **TNFSF4, **(C) **TNFRSF9, **(D) **KLRD1, **(E) **CD48, and **(F) **CD69). Data from two independent CD4+ and CD8+ T-cell experiments, E8 and E9, are shown.

Genes sharing similar expression patterns between CD4+ and CD8+ T cells, but different than CD3+ T cells, are likely important players in the communication between CD4+ and CD8+ compartments. Although not previously associated with T-cell activation, the decreased upregulation of NR4A2 (coactivator of general gene transcription) [13] and increased upregulation of IFI16 (transcriptional repressor) [14] and PSMB8, PSME2, and OTUB1(proteases) [15-17] are possibly involved in the delayed T-cell activation and proliferation of the CD4+ and CD8+ subsets compared to CD3+ T cells. The significant downregulation (at 48–96 hours) of IGLL1 and IGJ (Immunoglobulins), FCER1A (receptor), CD164 (negative regulator proliferation) and IRF9 (transcription factor) in CD3+ T cells, but not in CD4+ or CD8+ subset suggests that these proteins are affected by the co-presence and/or communication between the two subsets. Genes with different expression patterns between CD4+ and CD8+ subsets are possibly involved in cell-type-specific characteristics and functions. For instance, expression of TNFSF5 (CD40LG) has been mainly reported in CD4+ T cells, facilitating the activation of CD8+ T cells [18]. Indeed, a preferential transcriptional upregulation of TNFSF5 was observed in CD4+ T cells, supported by a protein abundance assay (Figure 4). Interestingly, TNFSF5 was also upregulated in CD8+ T cells (although not as strongly as in CD4+ T cells), supporting the recently reported expression of TNFSF5 in CD8+ T cells in the absence of CD4+ T cells [19]. This is an example demonstrating that, with the comparative analysis of the expression patterns among the 3 populations, our data capture significant differential transcriptional events. Transcriptional differences between CD4+ and CD8+ subsets were validated and supported by protein abundance assays of selected genes (TNFSF4, -5, -RSF9, KLRD1, CD48 and CD69) (Figure 4). Some of these genes (encoding cytokines and receptors) are discussed in detail below.

**Figure 5:**
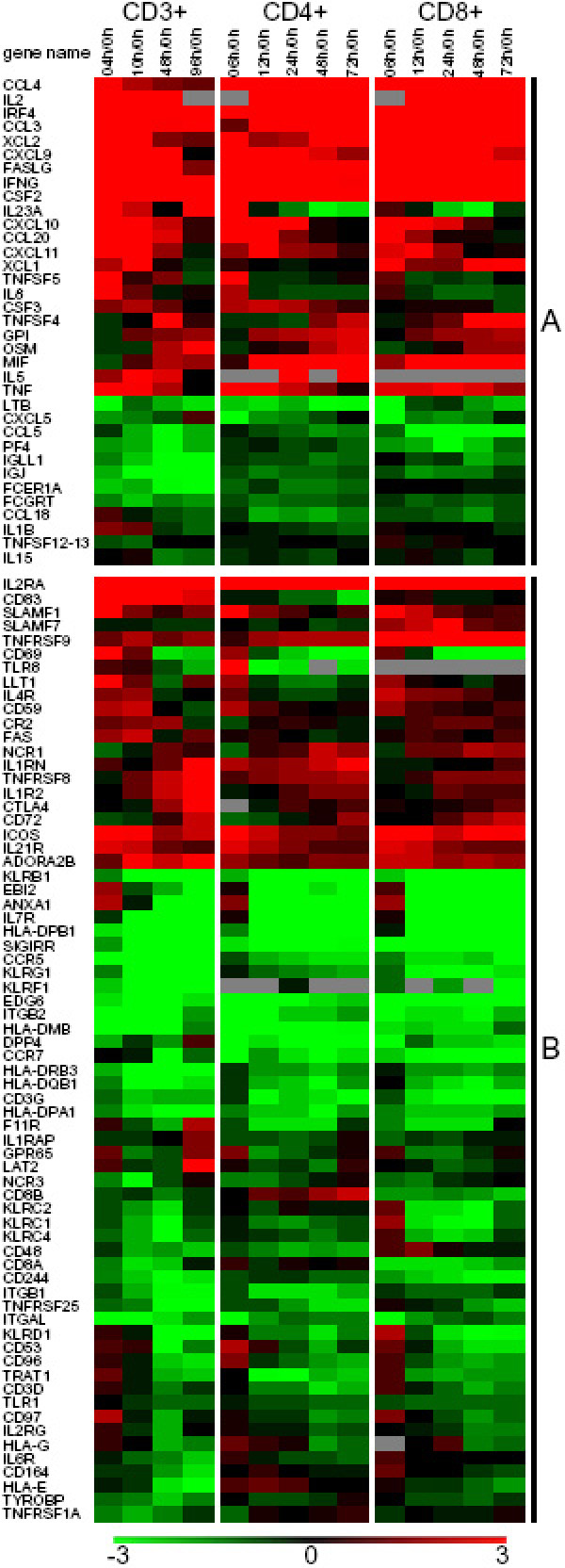
**Transcription profile of significant cytokines (A) and cytokine receptors (B)**. Cytokines and cytokine receptors, belonging to the 'immune response' Gene Ontology category, were sorted. Membership to these sets was manually curated from the corresponding gene pages in NCBI [20] and references therein. Color denotes degree of differential expression compared to 0 hour (saturated red = 3-fold up-regulation, saturated green = 3-fold down-regulation, black = unchanged, gray = no data available). Expression data shown are averages from three independent biological experiments for each T-cell population.

**Figure 6:**
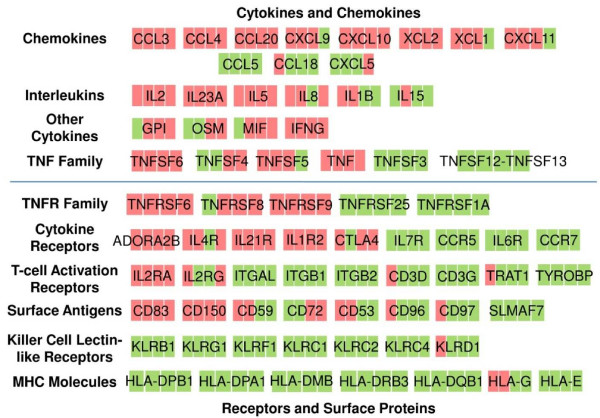
**Schematic showing the significantly regulated genes of cytokines and receptors**. Membership to these sets was manually curated from the corresponding gene pages in NCBI [20] and references therein. The regulation of gene transcription in CD3+ T cells, compared to 0 hour, is denoted by different color (green: downregulation; red: upregulation) at each timepoint in the sequence of 4, 10, 48 and 96 hours.

Cytokines act as messengers between cells, regulating their functions and activity. The production of cytokines is precisely controlled temporally in the immune response, and so are cytokine receptors. Thus, the significantly regulated cytokines and cytokine receptors in T-cell activation were sorted based on their functions listed by NCBI [20] (Figure 5 and Figure 6) and are discussed below.

### Cytokines and Chemokines

**Figure 7:**
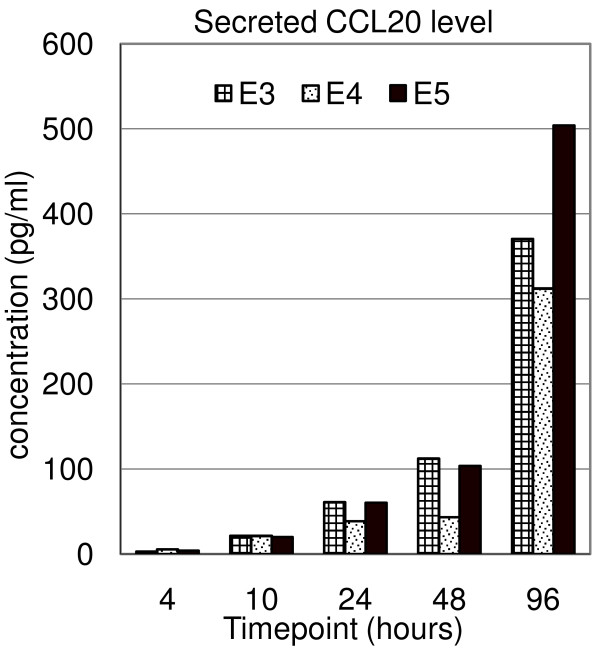
**Supernatant ELISA analysis of CCL20 secretion in three independent CD3+ T-cell experiments**. CD3+ T cells were selected, stimulated (by anti-CD3/anti-CD28 antibodies) and the supernatants were harvested at the indicated timepoints of culture. Data from three independent CD3+ T-cell experiments, E3–E5, are shown.

**Figure 8:**
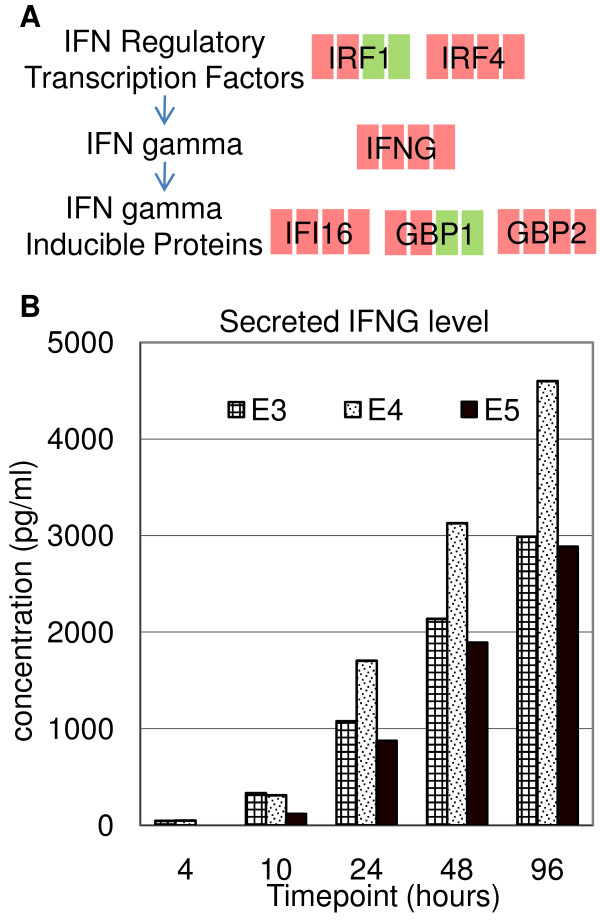
**Regulation of IFNG in T-cell activation**. (A) Schematic showing the significantly regulated genes associated with IFNG. The regulation of gene transcription in CD3+ T cells, compared to 0 hour, is denoted by different color (green: downregulation, red: upregulation) at each timepoint in the sequence of 4, 10, 48 and 96 hours. (B) Supernatant ELISA analysis of IFNG secretion in three independent CD3+ T-cell experiments, E3–E5. CD3+ T cells were selected, stimulated (by anti-CD3/anti-CD28 antibodies) and the supernatants were harvested at the indicated timepoints of culture.

A group of chemokines (CCL3, CCL4, CCL20, CXCL9, -10, -11, XCL1 and XCL2) showed a steady increasing expression upon anti-CD3/anti-CD28 stimulation in all 3 populations with the exception that XCL1 was not upregulated in CD4+ T cells. The strong upregulation of these genes is likely responsible for the proinflammatory response of T cells, including the recruitment of T cells as well as other leukocytes to the sites of inflammation. CCL20 is mainly secreted by epithelial cells and macrophages [21], and CXCL9, -10, -11 are mainly secreted by dendritic cells and macrophages [22,23]; their expressions have not been reported in T cells. Supernatant ELISA assays confirmed the significant continuous transcription upregulation of CCL20 (Figure 7), suggesting the induction of CCL20 secretion in T-cell activation. The preferential expression of XCL1 and XCL2 in CD8+ T cells suggests that these proteins might have roles in activation and/or functions of cytotoxic T cells [24]. A few chemokines showed high expression in resting T cells (CCL5, CCL18 and CXCL5), suggesting their importance in the homeostasis of resting T cells in the peripheral immune system. Interferon gamma (IFNG), INFG-inducible protein 16 (IFI16), IFN regulatory factor 4 (IRF4), -1, and IFNG-inducible Guanylate binding proteins (GBP1 and -2) were all upregulated (Figure 5 and Figure 8A). Supernatant ELISA assays revealed that the secreted IFNG protein level continuously increased throughout the 96 hours (Figure 8B). IFNG has important immunoregulatory functions such as antiviral and anti-tumor activity, and as an activator of macrophages [25], yet its functions in T-cell activation remains unknown. The orchestrated transcriptional regulation of IFN regulatory factors, IFNG, and INFG-inducible proteins and the significant induction of IFNG protein secretion suggest that IFNG secreted by T cells has an important role in T-cell activation.

Some of the interleukins (IL2, -23A, -5 and -8) were also upregulated, and with distinct patterns. The expression pattern of IL23A in CD3+ T cells was significantly different than that in CD4+ or CD8+ T cells. Expressed by activated dendritic cells, IL23 has anti-tumor effects through inducing CD4+ T-cell proliferation and its anti-tumor effects are reportedly to be inhibited by the depletion of CD4+ or CD8+ subset [26]. These expression differences among the three populations suggest that IL23A might be implicated in the communication between CD4+ and CD8+ T cells. Early upregulation of IL5 (reportedly a B-cell and eosinophil differentiation factor) [27] and IL8 (a neutrophil-activating factor) [28] suggests that they might have important roles in T cells as activating/differentiation factors. The downregulation (at 48–96 hours) of IL1B, mainly secreted by macrophages [29], and IL15, an important cytokine in lymphocyte survival [30], upon anti-CD3/anti-CD28 stimulation was unexpected.

A few members of the TNF family (TNFSF6 (FASLG), -4, -5 and TNF) were upregulated while others (LTB (TNFSF3) and TNFSF12-TNFSF13) were downregulated. A preferential expression in CD8+ T cells especially at 72 hours was observed for TNFSF4. TNFSF4 has been hypothesized to have costimulatory functions in both CD4+ and CD8+ subsets [31,32]. However, no subset-specific functions of TNFSF4 have been reported. Flow-cytometric analysis confirmed its significant upregulation at 72 hours in CD8+ T cells (Figure 4B). This preferential expression suggests that in CD8+ T cells, TNFSF4 might play an important role, possibly with cytotoxic effector functions besides the reported costimulatory functions.

A few cytokines (GPI, OSM and MIF) also displayed increased expression mainly at 10–96 hours. Neither the expression nor the function of these genes has been previously reported in T cells.

### Receptors

The upregulation of several key receptors (IL2RA (CD25), ICOS, and IL21R) was expected and confirms the validity of our data. However, the temporal expression patterns of these upregulated receptors, including those expressed throughout these experiments (IL2RA (CD25), ADORA2B), early (IL4R, IL21R, FAS) or late (IL1R2, TNFRSF8 and CTLA4), provide new insights that reflect their roles in T-cell activation. For instance, the late upregulation of CLTA4 is consistent with its inhibitory functions in T-cell activation [31]. The upregulation of FAS at 4–10 hours implies that FAS might have facilitating functions in early T-cell activation in addition to its known role in inducing apoptosis in fully activated cells [33]. The IL4 receptor is critical for inducing the development of the Th2 lineage of effector T cells [34]. The simultaneous early upregulation of IL4R and IL5 (a signature cytokine of Th2 cells) suggests that the Th1/Th2 balance might be biased towards the Th2 direction in our experiments. Significant differential expression of receptors, which have not been reported in T cells, call for attention to their possible role in T-cell activation. These include the upregulation of IL1R2 (as well as its binding proteins IL1RN and IL1RAP) at 96 hours and the constant upregulation of ADORA2B (G protein-coupled adenosine A2b receptor). Extensive upregulation of TNFRSF9 was observed in CD8+ subsets through 6–72 hours, but not in CD3+ T cells or CD4+ T cells. Flow cytometry analysis supported this preferential expression of TNFRSF9 in CD8+ T cells at the protein level (Figure 4C). TNFRSF9 has been reported as a costimulatory receptor in T cells, but not in a subset specific manner [25]. This continuous strong upregulation of TNFRSF9 in CD8+ T cells indicates its specific involvement in the activation, proliferation and differentiation of cytotoxic T cells.

Transcription of a number of other receptors, not previously reported in the context of T-cell action, was downregulated, likely to help achieve an efficient T-cell activation. These include IL7R (which shares the IL2 receptor gamma chain (IL2RG) with IL2RA) [35], CCR5, TNFRSF25 and TNFRSF1A (apoptosis inducing receptors) [36,37], CCR7 (enabling cells for secondary lymphoid organ homing) [38] and IL6R (regulating cell growth and differentiation in neutrophils) [39]. The unexpected downregulation of IL2RG (as opposed to the strong upregulated IL2RA) and of integrins (ITGAL, ITGB1 and ITGB2), the components of LFA-1 (reportedly a receptor for costimulatory signal in T-cell activation [40]), was somewhat surprising and deserves detailed attention.

Our data reveal the transcriptional dynamics of a few preciously reported upregulated cell-surface antigens including CD83 [41] (expressed throughout) and SLAMF1 (CD150) [42] (expressed early), and novel ones such as CD59 (expressed early) and CD72 (expressed late). Interestingly, CD83 showed increased expression upon stimulation in CD3+ T cells, decreased expression at 24–72 hours in CD4+ T cells, but no change in CD8+ T cells. CD83 expressed on dendritic cells delivers costimulatory signals to T cells for activation [43], and CD83 expressed on T cells has been hypothesized to be involved in T-cell activation with its detailed function yet to be defined [41]. These apparently different expression patterns among the three populations indicate that CD83 might have different roles in CD4+ versus CD8+ subsets, and may be possibly implicated in the communication of the subsets. In contrast to CD3+ or CD4+ T cells, SLAMF7 showed significant increased expression in CD8+ T cells at 6–24 hours. SLAMF7 regulates the cytotoxity of NK cells [44], and migration/adhesion of B cells [45]; however, its function in T cells has not been reported. This preferential expression of SLAMF7 in CD8+ T cells suggests that it might be involved in the development of the cytotoxicity of CD8+ T cells.

Among the mainly downregulated cell-surface antigens, the simultaneous downregulation of CD3D, CD3G, TRAT1 (TCR-associated transmembrane adaptor 1) and TYROBP (ZAP70 binding protein) along with the downregulation of LCK and ZAP70 (TCR associated tyrosine kinases) are likely part of the orchestrated regulation of T-cell activation. CD53, CD96, and CD97 shared similar expression patterns (upregulated at 4 hours and then downregulated at 48–96 hours). However, little is known about their roles in T-cell activation. We also observed the downregulation of CD8B and CD8A in CD8+ T-cell, possibly as a part of the CD8+ T-cell activation machinery. Interesting, binding partners, cell-surface antigens CD48 and CD244, demonstrated opposite expression patterns. CD48 was continuously downregulated in CD4+ T cells, but not in CD8+ T cells; while CD244 was continuously downregulated in CD8+ T cells, but not in CD4+ T cells. It has been hypothesized that T cells costimulate each other through the interactions of CD244 and CD48 [46]. This cell-type-specific downregulation of CD48 (CD4+ T-cell) and CD244 (CD8+ T-cell) suggests that this CD244–CD48 interaction might be cell-type-specific, between the CD244 expressing CD8+ T cells and CD48 expressing CD4+ T cells.

Upon activation, killer cell lectin-like receptors (KLRB1, KLRG1, KLRF1, KLRC1, -2, -4, and KLRD1) showed mainly decreased expression. First discovered in NK cells, the expression of inhibitory receptors (KLRG1, KLRB1, KLRC1, and KLRD1) [47,48], and activating receptor KLRC2 [49] has also been reported in activated CD8+ T cells, involved in TCR signalling, but not in activated CD4+ T cells or naive T cells. The shared downregulation of these KLRs along with that of some less-well-studied members (KLRF1 and KLRC4) suggests that their function might not be limited to effector CD8+ T cells, and that they are implicated more broadly in T-cell activation. Surprisingly, members of both MHC classes I and II were expressed in resting T cells (0 hour; data not shown). Several genes encoding MHC class II members (HLA-DPB1, HLA-DMB, HLA-DRB3, HLA-DQB1, and HLA-DPA1) showed decreased expression upon stimulation, while some MHC class I molecules (HLA-G and HLA-E) were downregulated at 48–96 hours.

### Granzymes

**Figure 9:**
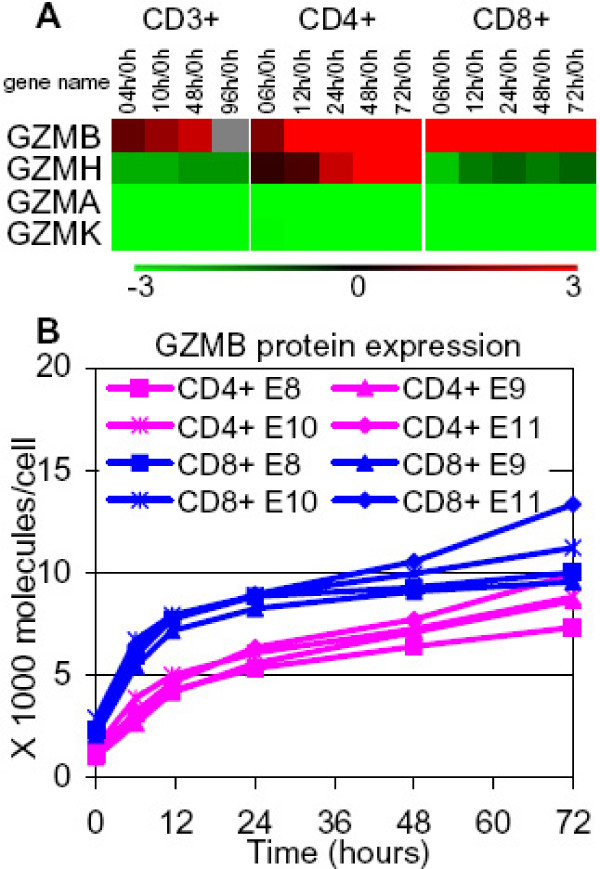
**GZMB was upregulated continuously in all three populations**. **(A) **Transcription profile of significantly regulated granzymes (GZMB, -A, -H, and -K). Color denotes degree of differential expression compared to 0 hour (saturated red = 3-fold up-regulation, saturated green = 3-fold down-regulation, black = unchanged, gray = no data available). Expression data shown are averages from three independent biological experiments for each T-cell population.**(B) **Intracellular protein expression profiles of GZMB in the CD4+ and CD8+ subsets. CD4+ T cells and CD8+ T cells were selected, stimulated (by anti-CD3/anti-CD28 antibodies), cultured separately and harvested at the indicated timepoints of culture to analyze the protein expression via by flow cytometric assays. Data from four independent CD4+ and CD8+ T-cell experiments, E8–E11, are shown.

Secretion of cytotoxic granules is one of the major effector functions of cytotoxic T cells to induce apoptosis in target cells [50]. Perforins, granulysin and granzymes are the core components of the dense cytotoxic granules responsible for target cell lysis. Most of the granzyme genes (GZMB, -A, -H, -K, except for GZMM), but not GNLY and PRF1, were significantly regulated (Figure 9A). GZMA and GZMK were considerably downregulated throughout the experiments. It is likely that T cells have not acquired the full cytotoxic effector functions at this stage of the activation. However, GZMB was transcriptionally upregulated in both CD4+ and CD8+ T cells, and this was supported by data from a protein abundance assay (Figure 9B).

## Discussion

Genome-scale transcriptional profiling can add significant new information for better understanding T-cell activation as an important biological process of the immune response. Previous efforts had examined T-cell activation at one single time point [8,9] or addressed costimulatory signal effects using only a single experiment [10,11]. Using multiple donors, in this study we focused on the temporal, donor-independent gene expression patterns not only in the CD3+ T cells, but also in the CD4+ and CD8+ subsets. We identified donor-independent significantly regulated genes in T-cell activation in CD3+ T cells (from the activation of co-cultures of CD4+ and CD8+ T cells in their natural ratio), and the CD4+ and CD8+ subsets. CD3+ T cells had far more significantly differentially expressed genes than CD4+ or CD8+ T cells (Figure 2). Regardless, the transcription profiles of the pooled significant genes in T-cell activation shared largely similar patterns among the three populations.

Anti-CD3/anti-CD28 stimulation induced expression pattern changes of 'immune response' genes which are consistent with the important roles of T cells in the adaptive immune response. Not only well-known cytokines (e.g., IL2, IFNG, TNFSF6 (FASLG)) and cytokine receptors (IL2RA (CD25), CD69, ICOS), but also numerous novel ones, for T-cell activation, were differentially expressed. Among the novel cytokines, the strongly upregulated chemokines (e.g., CCL3, -4, CCL20, CXCL9, -10, -11) might have important roles in enhancing T-cell activation in addition to their functions in cell trafficking, while the downregulated CCL5, IL5 might rather be involved in maintaining the homeostasis of resting T cells.

A number of cell-surface receptors not previously associated with T-cell activation, were differentially expressed, including downregulated CCR5, TNFRSF25, TNFRSF1A, CCR7 and IL6R, and upregulated CD59 and CD72. A number of the receptors involved in TCR activation (CD3D, CD3G, TRAT1, ITGAL, ITGB1, ITGB2, CD8A and CD8B (CD8+ T-cell specific) along with LCK, ZAP70 and TYROBP, were all simultaneously downregulated. Little is known about the expression and functions of KLRs in resting T cells. The downregulated KLR receptors are likely involved in the homeostasis of resting T cells. It is also possible that they regulate T-cell activation through TCR signalling. Surprisingly, both MHC class I and class II molecules were transcriptionally expressed in resting T cells (0 hour) (data not shown), and MHC class II molecules were significantly downregulated upon T-cell activation. MHC Class I and class II molecules are receptors on APCs, but not T cells, for the activation of CD8+ and CD4+ T cells, respectively.

Comparison of the expression patterns among the three populations provided further insights. Different expression patterns between CD4+ and CD8+ T cells (XCL1, -2, SLAMF7, CD244, CD48, TNFRSF9, TNFSF4, -5, CSF3, and GZMH) were observed, suggesting their subset specific involvement/functions in T-cell activation. Among these genes, very little is known about XCL2 and SLAMF7 in T cells. Genes (IL23A, NR4A2, CD83, PSME2, PSMB8, MIF) with similar expression patterns between CD4+ and CD8+ T cells, but different than those in CD3+ T cells are likely involved in the communication between CD4+ and CD8+ subsets. These and the large number of novel genes in the context of T-cell activation that were identified in this study offer new research targets for a more complete understanding of T-cell activation.

## Conclusion

Our study captured novel temporal patterns of previously known but many novel, in the context of T-cell activation, genes ontologically classified under the term 'immune response'. These patterns were reproducibly and robustly identified as donor independent, and were selectively confirmed by Q-RT-PCR and protein-level assays. Comprehensively integrating previous knowledge, we identified novel significant genes associated with the immune response in T cells, as well as subset specific genes, and genes implicated in the communication between CD4+ and CD8+ T cells. This study improves our understanding of the biology and the underlying regulation of T-cell activation in the natural CD3+ population, as well as in the CD4+ and CD8+ subsets.

## Methods

### Cells and culture system

Healthy-donor peripheral blood mononuclear cells (PBMCs) (AllCells, Berkeley, CA) were used to negatively-select CD3+ T cells, CD4+ and CD8+ T cells (Pan T Cell Isolation Kit II, CD4+ T Cell Isolation Kit II, and CD8+ T Cell Isolation Kit II, Miltenyi Biotech, Sunburn, CA). Cells were activated polyclonally with anti-CD3/anti-CD28 Mab (1:1)-coated magnetic beads (500 fmol/bead) (Dynabeads M-450 Epoxy, Dynal Biotech, Lake Success, NY) [51]. The ratio of beads to cells was 3:1. CD3+ cell cultures from three individual donor samples were seeded at 1 × 10^6 ^cells/mL in T-flasks and cultivated for 96 hours in serum-free AIM-V medium with 100 U/mL IL2 (Chiron, Emeryville, CA) and 2% human serum (Sigma-Aldrich St. Louis, MO) as described [52]. CD4+ cells and CD8+ cells from another three individual donors were cultured for 72 hours in the same manner. Cell counting and sampling for flow cytometry and microarray analysis were carried out at 0, 4, 10, 48 and 96 hours in CD3+ T-cell experiments, E1–E5, and at 0, 6, 12, 24, 48 and 72 hours in CD4+ T-cell and CD8+ T-cell experiments, E7–E11. This study was approved by the Northwestern University IRB.

### Flow cytometry

The following monoclonal antibodies (Mabs) for flow cytometry were purchased from BD Biosciences (San Jose, CA) unless otherwise stated and included CD3 (FITC+PE), CD4 PE, CD8 PE, CD25 PE, CD69 PE, TNFSF4 PE, CD40LG (TNFSF5) PE, TNFRSF9 PE, KLRD1 PE, CD48 PE, GZMB PE (Invitrogen, Carlsbad, CA). Flow cytometry was carried out as described [12,53]. Briefly, all samples were gated on forward scatter and on propidium iodide negative (PI-) to eliminate debris and dead cells. For intracellular detection of GZMB, cells were first stained with anti-CD4-FITC (or anti-CD8-FITC) and then fixed, permeabilized, and stained as previously described [54]. 10,000 gated events from each tube were acquired using a FACscan (BD Biosciences) or LSRII flow cytometer (Becton Dickinson). Quantibrite beads (BD Biosciences Immunocytometry Systems) labelled with different amounts of PE molecules were used to quantify surface or intracellular protein levels and normalize measurements between timepoints.

### RNA extraction and quality control

Total RNA was extracted from frozen cells using the Total RNA Isolation Mini Kit (Agilent, Wilmington, DE). RNA samples were re-suspended in RNase-free water and stored at -80°C. RNA yield and purity were assessed spectrophotometrically at 260 and 280 nm (Biomate 3, Thermo Spectronic, Marietta, OH). RNA integrity was evaluated using a Bioanalyzer 2100 (Agilent).

### DNA-microarray experiments and data analysis

Microarray-based transcriptional analysis was carried out for samples at each timepoint, using the 'reference' design [53], with Human Thymus Total RNA (Ambion, Austin, TX) as the reference RNA. Approximately half of the individual microarrays were replicated and the correlation coefficient between these technical replicates was above 0.90. Detailed experimental procedures and the use of the SNNLERM-algorithm [55] for data normalization were described [53]. Further analysis (significant genes identification, hierarchical clustering and gene ontology assignment) was carried out using 'MultiExperiment Viewer (MeV)' from The Institute for Genomic Research (TIGR) [56]. Raw and normalized data were deposited in the Gene Expression Omnibus (GSE6607 (CD3+ T-cell experiment), GSE7571 (CD4+ T-cell experiment) and GSE7572 (CD8+ T-cell experiment)) [57]. Within each population (three biological replicates using cells from different donors), multi-class SAM (Significance Analysis of Microarrays) with a false discovery rate <1% was used to select genes that show statistically different expression between groups. A group is defined as all the samples belonging to the same timepoint regardless of the donors. Briefly, there were 5 groups (0 hour, 4, 10, 48 and 96 hours) in the set of CD3+ experiments, E1–E3, and 6 groups (0 hour, 6, 12, 24, 48 and 72 hours) in the set of CD4+ experiments and CD8+ experiments, E7–E9. The three samples from biological experiments in each group were treated as replicates. To focus on the expression change, gene expression at each time point was compared to that of 0 hour in each experiment. Gene Ontology annotations, as curated by European Bioinformatics Institute, were retrieved from the Gene Ontology Consortium website [58]. Hierarchical clustering was performed with the Euclidian distance metric.

### Quantitative RT-PCR (Q-RT-PCR)

cDNA was obtained from total RNA samples using the High-Capacity cDNA Archive Kit and Q-RT-PCR was performed with Assays-on-Demand kits (Applied Biosystems; Foster City, CA) as described [12]. The amount of mRNA for each sample was normalized using the average of two housekeeping genes (Glucuronidase-β and 18S). The use of GUSB (Hs99999908_m1) and 18S (Hs99999901_s1) genes as housekeeping genes has been previously tested in our lab [12,53]. Primers (Applied Biosystems, Foster City, CA) for the following functionally diverse set of genes were used: FOS (Hs01119267_g1), MYB (Hs00920564_m1), JUN (Hs99999141_s1), CAT (Hs00156308_m1), MAPK6 (Hs00957318_g1), SOD2 (Hs00167309_m1), SORD (Hs00973148_m1), STAT1 (Hs01014001_m1) in CD3+ T-cell experiments, E1–E3; and GZMA (Hs00196206_m1), GZMB (Hs00188051_m1), MYB (Hs00920564_m1), FASLG (Hs00899442_m1), EGR1 (Hs00152928_m1), EGR2 (Hs00166165_m1), and EGR3 (Hs00231780_m1) in CD4+ and CD8+ T-cell experiments, E8 and E9. Genes were chosen to reflect differentially expressed genes of a wide range of microarray signal intensities.

### Supernatant ELISA assay of CCL20 and IFNG

Culture supernatants were collected at 4, 10, 48 and 96 hours in three CD3+ T-cell experiments, E3–E5, and analyzed for concentrations of CCL20 and IFNG by ELISA (R&D Systems, Minneapolis) following the manufacturer's instructions.

## Abbreviations

PBMC: peripheral blood mononuclear cell; MHC: major histocompatibility complex; APC: antigen-presenting cell; TCR: T-cell receptor; HLA: human leukocyte antigen; KLR: killer cell lectin-like receptor; LFA:  lymphocyte function-associated antigen; GO: Gene Ontology; TNF: tumor necrosis factor; NK cells: natural killer cells; Mab: monoclonal antibody; PI: propidium iodide; FITC: fluorescein isothiocyanate; PE:  phycoerythrin; Q-RT-PCR: quantitative reverse-transcription polymerase chain reaction.

## Authors' contributions

MW and DW carried out the culture experiments and performed the microarray experiments. MW analyzed the microarray data and wrote the manuscript with assistance from ETP. MW and ETP conceived, designed, and coordinated the study.

## Supplementary Material

Additional file 1 Reproducibility of expression profiles of the T-cell activation in CD3+ cells. Hierarchical clustering (using the Euclidian distance metric) of the 3793 significant genes in T-cell activation of CD3+ cells in three independent biological experiments, E1–E3, (timepoints at 4, 10, 48 and 96 hours) demonstrated high reproducibility. Color denotes degree of differential expression compared to 0 hour (saturated red = 3-fold upregulation, saturated green = 3-fold down-regulation, black = unchanged, gray = no data available). Click here for file

Additional file 2 Complete list of the 3793 significant genes in T-cell activation of CD3+ cells in three independent biological experiments, E1–E3. Color denotes degree of differential expression compared to 0 hour (saturated red = 3-fold upregulation, saturated green = 3-fold down-regulation, black = unchanged, gray = no data available). Click here for file

Additional file 3 Reproducibility of expression profiles of the T-cell activation in CD4+ cells. Hierarchical clustering (using the Euclidian distance metric) of the 1463 significant genes in T-cell activation of CD4+ cells in three independent biological experiments (timepoints at 12, 24, 48 and 72 hours in one experiment, E7; and timepoints at 6, 12, 24, 48 and 72 hours in the other two experiments, E8 and E9) demonstrated high reproducibility. Color denotes degree of differential expression compared to 0 hour (saturated red = 3-fold upregulation, saturated green = 3-fold down-regulation, black = unchanged, gray = no data available). Click here for file

Additional file 4 Complete list of the 1463 significant genes in T-cell activation of CD4+ cells in three independent biological experiments, E7–E9. Color denotes degree of differential expression compared to 0 hour (saturated red = 3-fold upregulation, saturated green = 3-fold down-regulation, black = unchanged, gray = no data available). Click here for file

Additional file 5 Reproducibility of expression profiles of the T-cell activation in CD8+ cells. Hierarchical clustering (using the Euclidian distance metric) of the 1258 significant genes in T-cell activation of CD8+ cells in three independent biological experiments (timepoints at 12, 24, 48 and 72 hours in one experiment, E7; and timepoints at 6, 12, 24, 48 and 72 hours in the other two experiments, E8 and E9) demonstrated high reproducibility. Color denotes degree of differential expression compared to 0 hour (saturated red = 3-fold upregulation, saturated green = 3-fold down-regulation, black = unchanged, gray = no data available). Click here for file

Additional file 6 Complete list of the 1258 significant genes in T-cell activation of CD8+ cells in three independent biological experiments, E7–E9. Color denotes degree of differential expression compared to 0 hour (saturated red = 3-fold upregulation, saturated green = 3-fold down-regulation, black = unchanged, gray = no data available). Click here for file

Additional file 7 The three populations, CD3+, CD4+ and CD8+ T cells, shared largely conserved expression patterns for the significant genes, demonstrated by the hierarchical clustering (using the Euclidian distance metric) of the combined 4167 significant genes upon T-cell activation in CD3+, CD4+ and CD8+ T-cell populations (average of three biological-replicate experiments for each population). Color denotes degree of differential expression comparing to 0 hour (saturated red = 3-fold upregulation, saturated green = 3-fold down-regulation, black = unchanged, gray = no data available). Click here for file

Additional file 8 Complete list of the combined 4167 significant genes upon T-cell activation in CD3+, CD4+ and CD8+ T-cell populations (average of three biological-replicate experiments for each population). Color denotes degree of differential expression comparing to 0 hour (saturated red = 3-fold upregulation, saturated green = 3-fold down-regulation, black = unchanged, gray = no data available). Click here for file

Additional file 9 Q-RT-PCR validation of microarray results across multiple culture samples. **(A) **Q-RT-PCR versus microarray log expression ratios (timepoint vs. 0 hour) from CD3+ T-cell activation experiments, E1–E3, (for all 12 (= 3 × 4) timepoints: 4, 10, 48 and 96 hours of 3 experiments) for each of the 8 selected genes (FOS, MYB, JUN, CAT, MAPK6, SORD, SOD2, and STAT1). (**B) **Q-RT-PCR versus microarray log expression ratios (timepoint vs. 0 hour) from CD4+ and CD8+ T-cell activation experiments, E8 and E9, (for all 20 (= 2 × 2 × 5) timepoints: 6, 12, 24, 48 and 72 hours of 2 experiments) for each of the 7 selected genes (EGR1, EGR2, EGR3, FASL, GZMA, GZMB, and MYB). Click here for file

## References

[B1] Palmer E (2003). Negative selection--clearing out the bad apples from the T-cell repertoire. Nat Rev Immunol.

[B2] Santana MA, Rosenstein Y (2003). What it takes to become an effector T cell: the process, the cells involved, and the mechanisms. J Cell Physiol.

[B3] Mellman I, Steinman RM (2001). Dendritic cells: specialized and regulated antigen processing machines. Cell.

[B4] Rothstein DM, Sayegh MH (2003). T-cell costimulatory pathways in allograft rejection and tolerance. Immunol Rev.

[B5] Huang Y, Wange RL (2004). T cell receptor signaling: beyond complex complexes. J Biol Chem.

[B6] Germain RN, Stefanova I (1999). The dynamics of T cell receptor signaling: complex orchestration and the key roles of tempo and cooperation. Annu Rev Immunol.

[B7] Kemp ML, Wille L, Lewis CL, Nicholson LB, Lauffenburger DA (2007). Quantitative Network Signal Combinations Downstream of TCR Activation Can Predict IL-2 Production Response. J Immunol.

[B8] Chtanova T, Newton R, Liu SM, Weininger L, Young TR, Silva DG, Bertoni F, Rinaldi A, Chappaz S, Sallusto F, Rolph MS, Mackay CR (2005). Identification of T cell-restricted genes, and signatures for different T cell responses, using a comprehensive collection of microarray datasets. J Immunol.

[B9] Hughes-Fulford M, Sugano E, Schopper T, Li CF, Boonyaratanakornkit JB, Cogoli A (2005). Early immune response and regulation of IL-2 receptor subunits. Cell Signal.

[B10] Diehn M, Alizadeh AA, Rando OJ, Liu CL, Stankunas K, Botstein D, Crabtree GR, Brown PO (2002). Genomic expression programs and the integration of the CD28 costimulatory signal in T cell activation. Proc Natl Acad Sci U S A.

[B11] Riley JL, Mao M, Kobayashi S, Biery M, Burchard J, Cavet G, Gregson BP, June CH, Linsley PS (2002). Modulation of TCR-induced transcriptional profiles by ligation of CD28, ICOS, and CTLA-4 receptors. Proc Natl Acad Sci U S A.

[B12] Fuhrken PG, Chen C, Miller WM, Papoutsakis ET (2007). Comparative, genome-scale transcriptional analysis of CHRF-288-11 and primary human megakaryocytic cell cultures provides novel insights into lineage-specific differentiation. Exp Hematol.

[B13] Paulsen RF, Granas K, Johnsen H, Rolseth V, Sterri S (1995). Three related brain nuclear receptors, NGFI-B, Nurr1, and NOR-1, as transcriptional activators. J Mol Neurosci.

[B14] Johnstone RW, Kerry JA, Trapani JA (1998). The human interferon-inducible protein, IFI 16, is a repressor of transcription. J Biol Chem.

[B15] De M, Jayarapu K, Elenich L, Monaco JJ, Colbert RA, Griffin TA (2003). Beta 2 subunit propeptides influence cooperative proteasome assembly. J Biol Chem.

[B16] Heink S, Ludwig D, Kloetzel PM, Kruger E (2005). IFN-gamma-induced immune adaptation of the proteasome system is an accelerated and transient response. Proc Natl Acad Sci U S A.

[B17] Soares L, Seroogy C, Skrenta H, Anandasabapathy N, Lovelace P, Chung CD, Engleman E, Fathman CG (2004). Two isoforms of otubain 1 regulate T cell anergy via GRAIL. Nat Immunol.

[B18] Bourgeois C, Rocha B, Tanchot C (2002). A role for CD40 expression on CD8+ T cells in the generation of CD8+ T cell memory. Science.

[B19] Hernandez MG, Shen L, Rock KL (2007). CD40-CD40 ligand interaction between dendritic cells and CD8+ T cells is needed to stimulate maximal T cell responses in the absence of CD4+ T cell help. J Immunol.

[B20] The National Center for Biotechnology Information Website. http://www.ncbi.nlm.nih.gov/.

[B21] Fitzhugh DJ, Naik S, Caughman SW, Hwang ST (2000). Cutting edge: C-C chemokine receptor 6 is essential for arrest of a subset of memory T cells on activated dermal microvascular endothelial cells under physiologic flow conditions in vitro. J Immunol.

[B22] Lasagni L, Francalanci M, Annunziato F, Lazzeri E, Giannini S, Cosmi L, Sagrinati C, Mazzinghi B, Orlando C, Maggi E, Marra F, Romagnani S, Serio M, Romagnani P (2003). An alternatively spliced variant of CXCR3 mediates the inhibition of endothelial cell growth induced by IP-10, Mig, and I-TAC, and acts as functional receptor for platelet factor 4. J Exp Med.

[B23] Luster AD (2002). The role of chemokines in linking innate and adaptive immunity. Curr Opin Immunol.

[B24] Wilkinson J, Zaunders JJ, Carr A, Guillemin G, Cooper DA (2002). Characterization of the phenotypic and lymphokine profile associated with strong CD8+ anti-HIV-1 suppressor activity (CASA). Clin Exp Immunol.

[B25] Schroder K, Hertzog PJ, Ravasi T, Hume DA (2004). Interferon-gamma: an overview of signals, mechanisms and functions. J Leukoc Biol.

[B26] Kaiga T, Sato M, Kaneda H, Iwakura Y, Takayama T, Tahara H (2007). Systemic administration of IL-23 induces potent antitumor immunity primarily mediated through Th1-type response in association with the endogenously expressed IL-12. J Immunol.

[B27] Geijsen N, Koenderman L, Coffer PJ (2001). Specificity in cytokine signal transduction: lessons learned from the IL-3/IL-5/GM-CSF receptor family. Cytokine Growth Factor Rev.

[B28] Drost EM, MacNee W (2002). Potential role of IL-8, platelet-activating factor and TNF-alpha in the sequestration of neutrophils in the lung: effects on neutrophil deformability, adhesion receptor expression, and chemotaxis. Eur J Immunol.

[B29] Basak C, Pathak SK, Bhattacharyya A, Mandal D, Pathak S, Kundu M (2005). NF-kappaB- and C/EBPbeta-driven interleukin-1beta gene expression and PAK1-mediated caspase-1 activation play essential roles in interleukin-1beta release from Helicobacter pylori lipopolysaccharide-stimulated macrophages. J Biol Chem.

[B30] Eidenschenk C, Jouanguy E, Alcais A, Mention JJ, Pasquier B, Fleckenstein IM, Puel A, Gineau L, Carel JC, Vivier E, Le Deist F, Casanova JL (2006). Familial NK cell deficiency associated with impaired IL-2- and IL-15-dependent survival of lymphocytes. J Immunol.

[B31] Manzotti CN, Tipping H, Perry LC, Mead KI, Blair PJ, Zheng Y, Sansom DM (2002). Inhibition of human T cell proliferation by CTLA-4 utilizes CD80 and requires CD25+ regulatory T cells. Eur J Immunol.

[B32] Serghides L, Bukczynski J, Wen T, Wang C, Routy JP, Boulassel MR, Sekaly RP, Ostrowski M, Bernard NF, Watts TH (2005). Evaluation of OX40 ligand as a costimulator of human antiviral memory CD8 T cell responses: comparison with B7.1 and 4-1BBL. J Immunol.

[B33] Davidson WF, Haudenschild C, Kwon J, Williams MS (2002). T cell receptor ligation triggers novel nonapoptotic cell death pathways that are Fas-independent or Fas-dependent. J Immunol.

[B34] Mora AL, Stephenson LM, Enerson B, Youn J, Keegan AD, Boothby M (2003). New programming of IL-4 receptor signal transduction in activated T cells: Stat6 induction and Th2 differentiation mediated by IL-4Ralpha lacking cytoplasmic tyrosines. J Immunol.

[B35] Xue HH, Kovanen PE, Pise-Masison CA, Berg M, Radovich MF, Brady JN, Leonard WJ (2002). IL-2 negatively regulates IL-7 receptor alpha chain expression in activated T lymphocytes. Proc Natl Acad Sci U S A.

[B36] Jiang Y, Woronicz JD, Liu W, Goeddel DV (1999). Prevention of constitutive TNF receptor 1 signaling by silencer of death domains. Science.

[B37] Murooka TT, Wong MM, Rahbar R, Majchrzak-Kita B, Proudfoot AE, Fish EN (2006). CCL5-CCR5-mediated apoptosis in T cells: Requirement for glycosaminoglycan binding and CCL5 aggregation. J Biol Chem.

[B38] Fritsch RD, Shen X, Sims GP, Hathcock KS, Hodes RJ, Lipsky PE (2005). Stepwise differentiation of CD4 memory T cells defined by expression of CCR7 and CD27. J Immunol.

[B39] Walker F, Zhang HH, Matthews V, Weinstock J, Nice EC, Ernst M, Rose-John S, Burgess AW (2007). IL6/sIL6R complex contributes to emergency granulopoietic response in G-CSF and GM-CSF deficient mice. Blood.

[B40] Mor A, Campi G, Du G, Zheng Y, Foster DA, Dustin ML, Philips MR (2007). The lymphocyte function-associated antigen-1 receptor costimulates plasma membrane Ras via phospholipase D2. Nat Cell Biol.

[B41] Wolenski M, Cramer SO, Ehrlich S, Steeg C, Fleischer B, von Bonin A (2003). Enhanced activation of CD83-positive T cells. Scand J Immunol.

[B42] Cocks BG, Chang CC, Carballido JM, Yssel H, de Vries JE, Aversa G (1995). A novel receptor involved in T-cell activation. Nature.

[B43] Aerts-Toegaert C, Heirman C, Tuyaerts S, Corthals J, Aerts JL, Bonehill A, Thielemans K, Breckpot K (2007). CD83 expression on dendritic cells and T cells: correlation with effective immune responses. Eur J Immunol.

[B44] Lee JK, Boles KS, Mathew PA (2004). Molecular and functional characterization of a CS1 (CRACC) splice variant expressed in human NK cells that does not contain immunoreceptor tyrosine-based switch motifs. Eur J Immunol.

[B45] Lee JK, Mathew SO, Vaidya SV, Kumaresan PR, Mathew PA (2007). CS1 (CRACC, CD319) induces proliferation and autocrine cytokine expression on human B lymphocytes. J Immunol.

[B46] Assarsson E, Kambayashi T, Persson CM, Chambers BJ, Ljunggren HG (2005). 2B4/CD48-mediated regulation of lymphocyte activation and function. J Immunol.

[B47] Mingari MC, Ponte M, Vitale C, Bellomo R, Moretta L (2000). Expression of HLA class I-specific inhibitory receptors in human cytolytic T lymphocytes: a regulated mechanism that controls T-cell activation and function. Hum Immunol.

[B48] Robbins SH, Nguyen KB, Takahashi N, Mikayama T, Biron CA, Brossay L (2002). Cutting edge: inhibitory functions of the killer cell lectin-like receptor G1 molecule during the activation of mouse NK cells. J Immunol.

[B49] Arlettaz L, Villard J, de Rham C, Degermann S, Chapuis B, Huard B, Roosnek E (2004). Activating CD94:NKG2C and inhibitory CD94:NKG2A receptors are expressed by distinct subsets of committed CD8+ TCR alphabeta lymphocytes. Eur J Immunol.

[B50] Andersen MH, Schrama D, Thor Straten P, Becker JC (2006). Cytotoxic T cells. J Invest Dermatol.

[B51] Ito F, Carr A, Svensson H, Yu J, Chang AE, Li Q (2003). Antitumor reactivity of anti-CD3/anti-CD28 bead-activated lymphoid cells: implications for cell therapy in a murine model. J Immunother.

[B52] Eisen MB, Spellman PT, Brown PO, Botstein D (1998). Cluster analysis and display of genome-wide expression patterns. Proc Natl Acad Sci U S A.

[B53] Ramsborg CG, Papoutsakis ET (2007). Global transcriptional analysis delineates the differential inflammatory response interleukin-15 elicits from cultured human T cells. Exp Hematol.

[B54] Ramsborg CG, Windgassen D, Fallon JK, Paredes CJ, Papoutsakis ET (2004). Molecular insights into the pleiotropic effects of plasma on ex vivo-expanded T cells using DNA-microarray analysis. Exp Hematol.

[B55] Yang H, Haddad H, Tomas C, Alsaker K, Papoutsakis ET (2003). A segmental nearest neighbor normalization and gene identification method gives superior results for DNA-array analysis. Proc Natl Acad Sci U S A.

[B56] Saeed AI, Sharov V, White J, Li J, Liang W, Bhagabati N, Braisted J, Klapa M, Currier T, Thiagarajan M, Sturn A, Snuffin M, Rezantsev A, Popov D, Ryltsov A, Kostukovich E, Borisovsky I, Liu Z, Vinsavich A, Trush V, Quackenbush J (2003). TM4: a free, open-source system for microarray data management and analysis. Biotechniques.

[B57] The Gene Expression Omnibus. http://www.ncbi.nlm.nih.gov/geo/.

[B58] The Gene Ontology Consortium Website. http://www.geneontology.org/.

