# Supplementary figures and images for: Comparative analysis of transcriptional profiling of CD3+, CD4+ and CD8+ T cells identifies novel immune response players in T-Cell activation
Source: BMC Genomics. 2008 May 16;9:225. doi: 10.1186/1471-2164-9-225 (PMC2396644; doi:10.1186/1471-2164-9-225)

E1

04h/0h 10h/0h 48h/0h 96h/0h

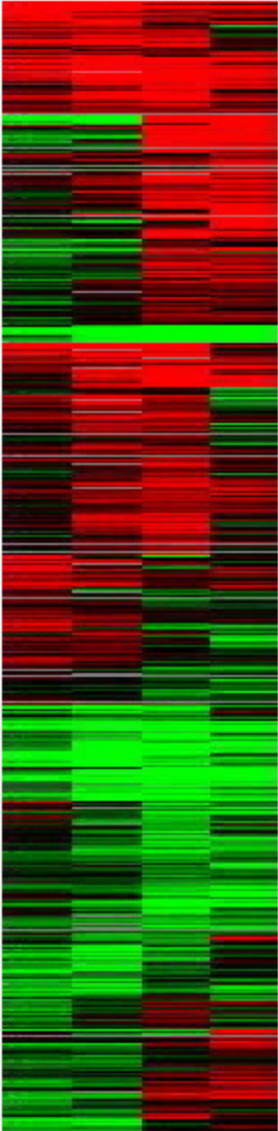

E2

04h/0h 10h/0h 48h/0h 96h/0h

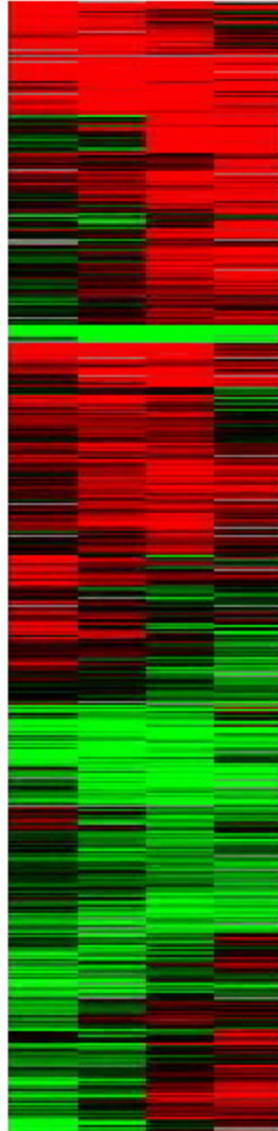

E3

04h/0h 10h/0h 48h/0h 96h/0h

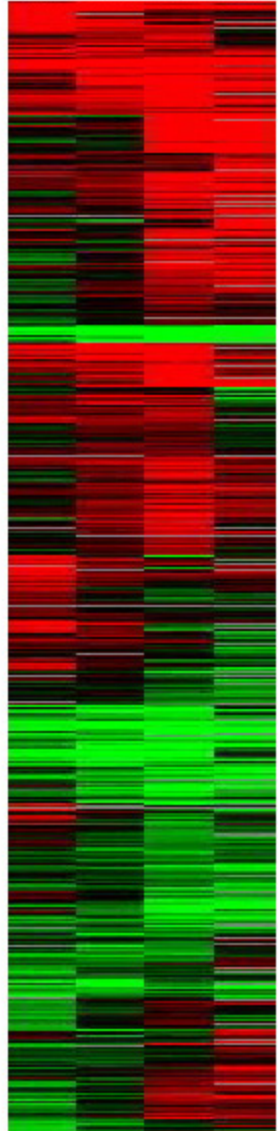

-3 0 3

Supplement: Additional file 1 — Reproducibility of expression profiles of the T-cell activation in CD3+ cells. Hierarchical clustering (using the Euclidian distance metric) of the 3793 significant genes in T-cell activation of CD3+ cells in three independent biological experiments, E1–E3, (timepoints at 4, 10, 48 and 96 hours) demonstrated high reproducibility. Color denotes degree of differential expression compared to 0 hour (saturated red = 3-fold upregulation, saturated green = 3-fold down-regulation, black = unchanged, gray = no data available). [file 1471-2164-9-225-S1.pdf]

**E7**

12h/0h 24h/0h 48h/0h 72h/0h

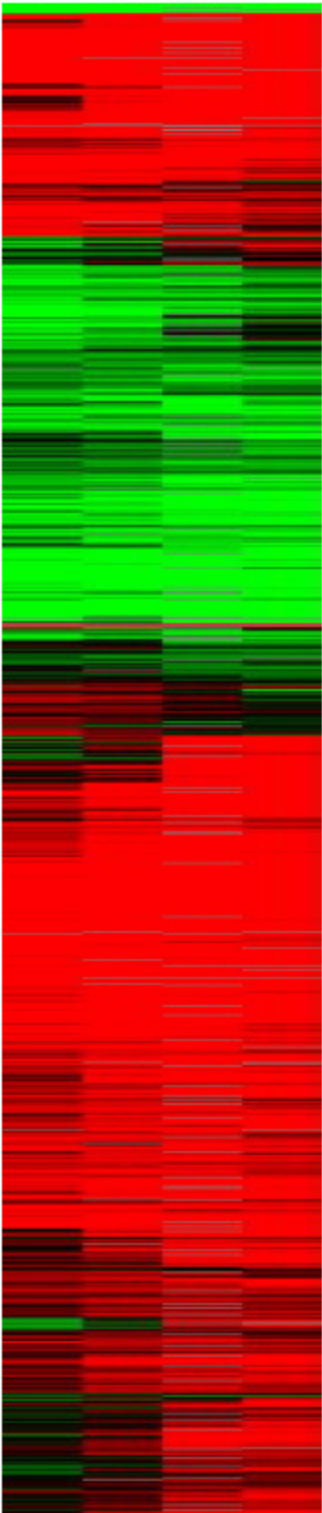**E8**

06h/0h 12h/0h 24h/0h 48h/0h 72h/0h

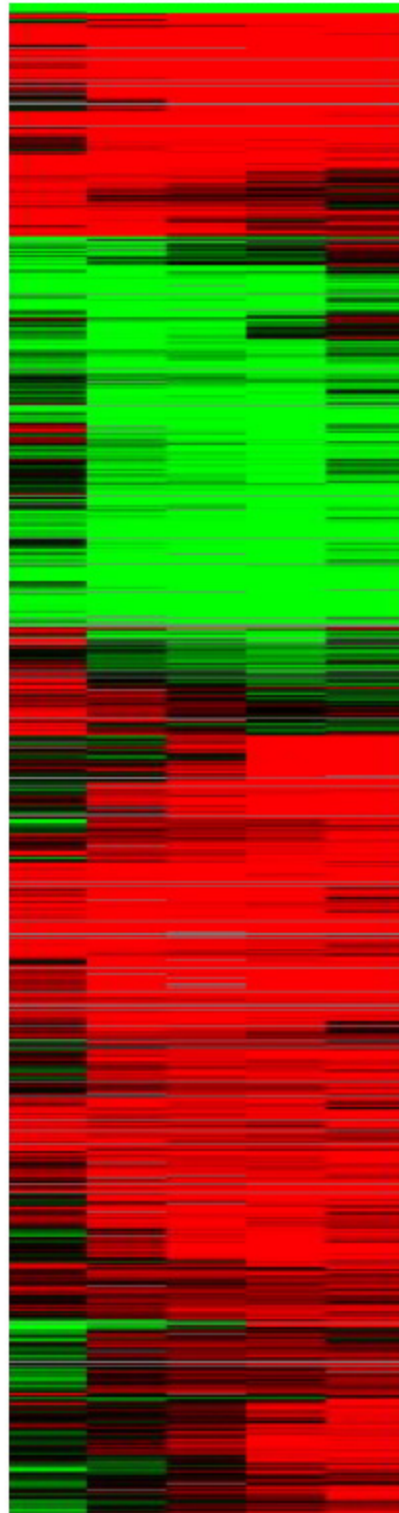**E9**

06h/0h 12h/0h 24h/0h 48h/0h 72h/0h

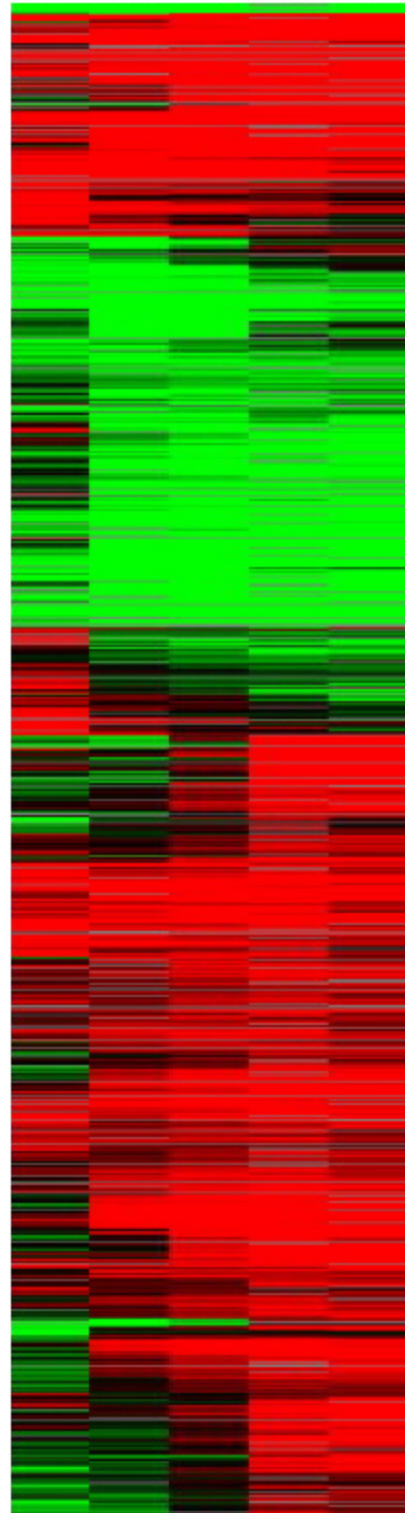

-3 0 3

Supplement: Additional file 3 — Reproducibility of expression profiles of the T-cell activation in CD4+ cells. Hierarchical clustering (using the Euclidian distance metric) of the 1463 significant genes in T-cell activation of CD4+ cells in three independent biological experiments (timepoints at 12, 24, 48 and 72 hours in one experiment, E7; and timepoints at 6, 12, 24, 48 and 72 hours in the other two experiments, E8 and E9) demonstrated high reproducibility. Color denotes degree of differential expression compared to 0 hour (saturated red = 3-fold upregulation, saturated green = 3-fold down-regulation, black = unchanged, gray = no data available). [file 1471-2164-9-225-S3.pdf]

**E7**

12h/0h 24h/0h 48h/0h 72h/0h

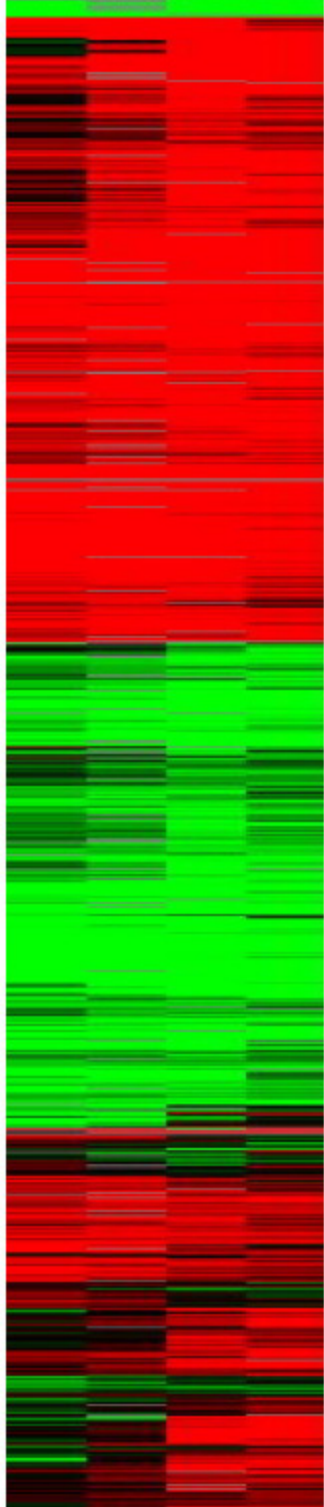**E8**

06h/0h 12h/0h 24h/0h 48h/0h 72h/0h

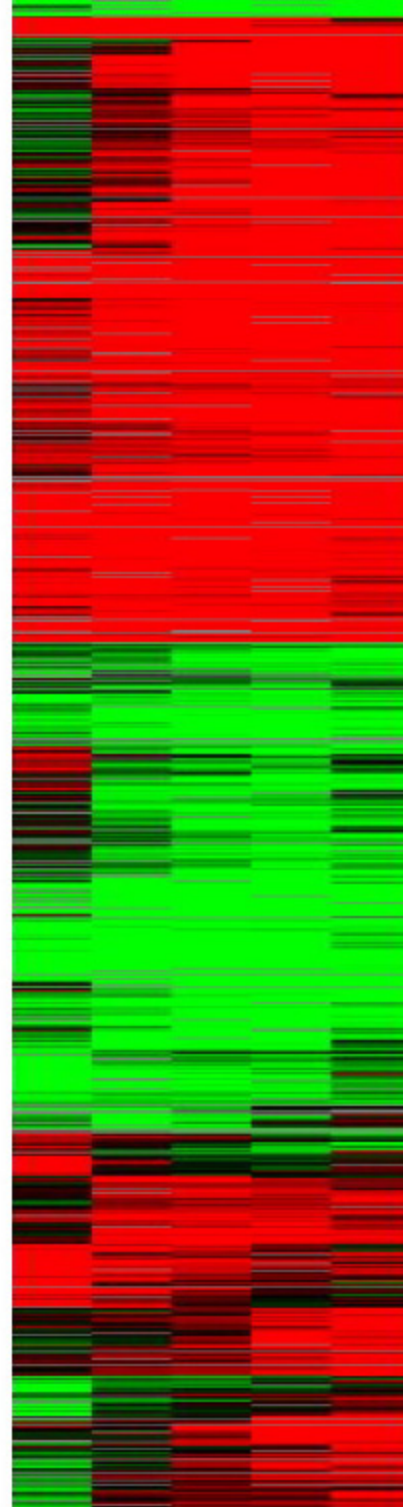**E9**

06h/0h 12h/0h 24h/0h 48h/0h 72h/0h

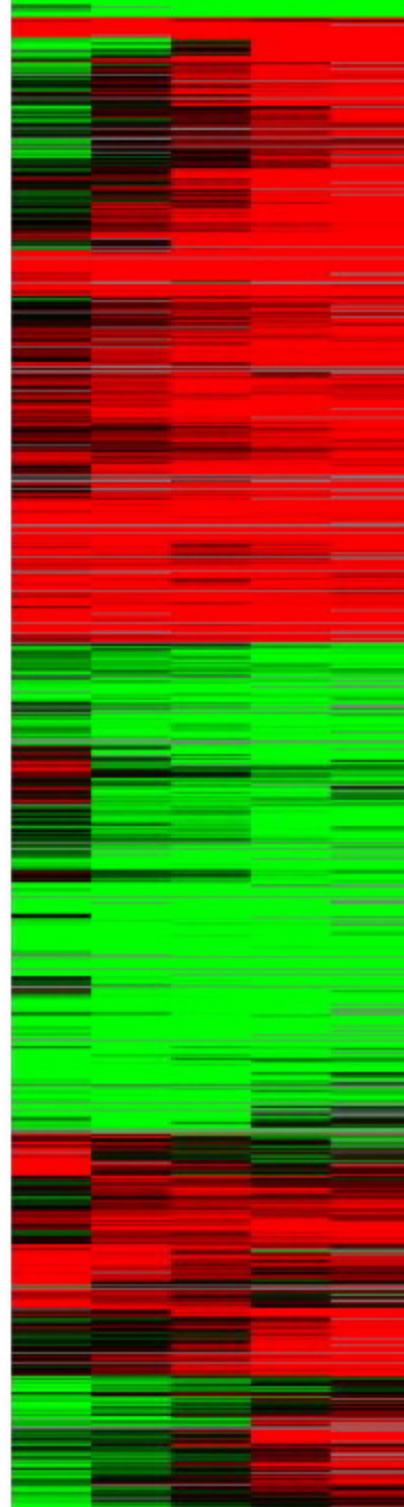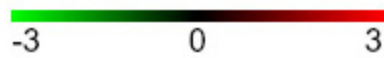

Supplement: Additional file 5 — Reproducibility of expression profiles of the T-cell activation in CD8+ cells. Hierarchical clustering (using the Euclidian distance metric) of the 1258 significant genes in T-cell activation of CD8+ cells in three independent biological experiments (timepoints at 12, 24, 48 and 72 hours in one experiment, E7; and timepoints at 6, 12, 24, 48 and 72 hours in the other two experiments, E8 and E9) demonstrated high reproducibility. Color denotes degree of differential expression compared to 0 hour (saturated red = 3-fold upregulation, saturated green = 3-fold down-regulation, black = unchanged, gray = no data available). [file 1471-2164-9-225-S5.pdf]

**CD3+**

04h/0h 10h/0h 48h/0h 96h/0h

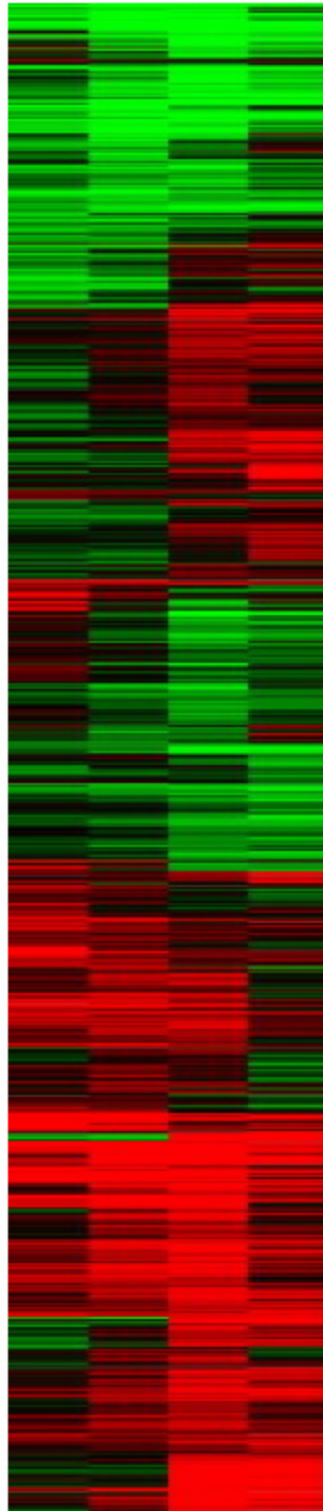**CD4+**

06h/0h 12h/0h 24h/0h 48h/0h 72h/0h

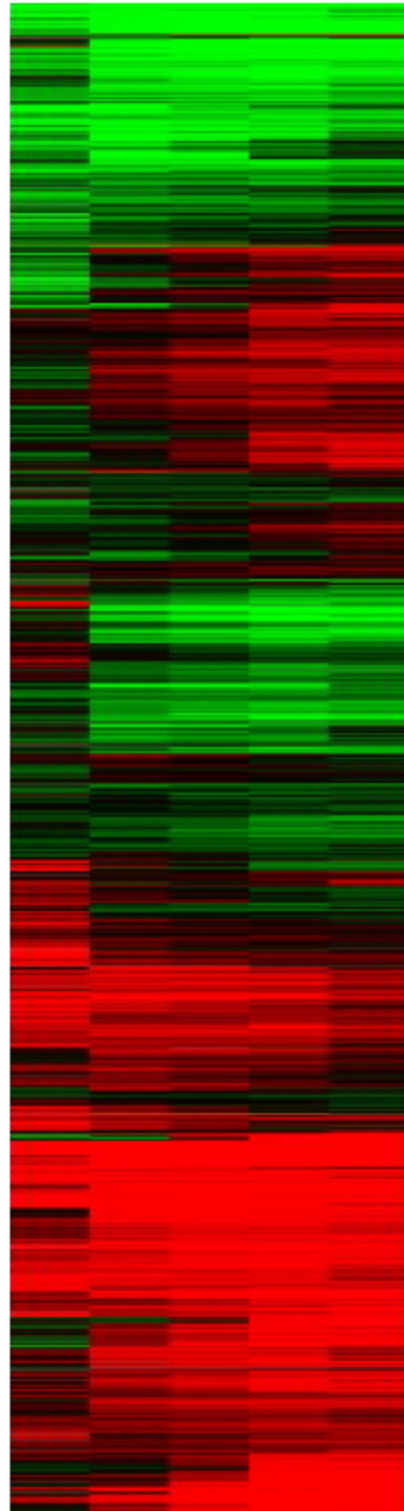**CD8+**

06h/0h 12h/0h 24h/0h 48h/0h 72h/0h

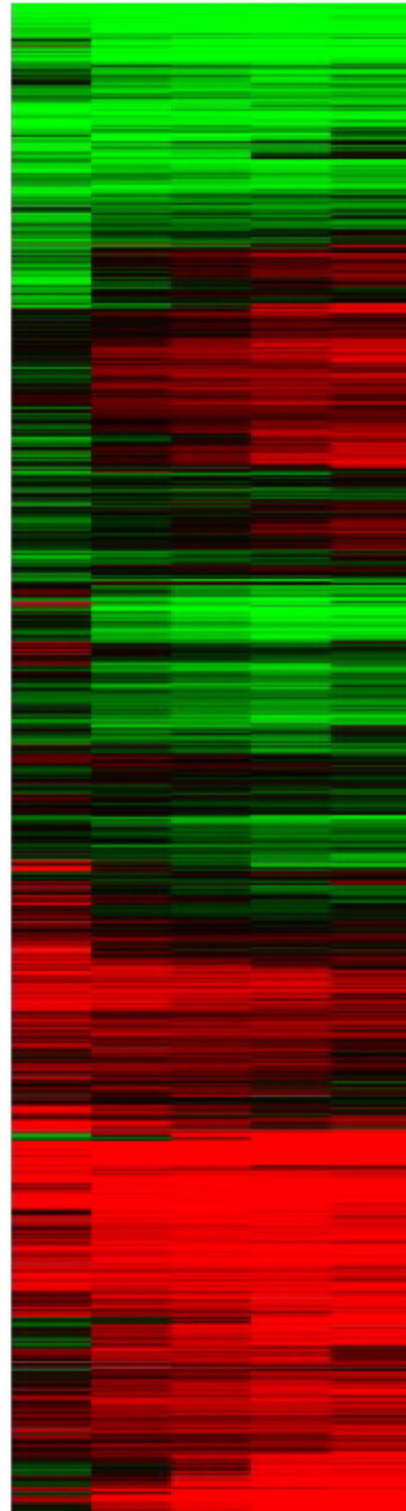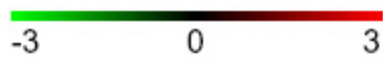

Supplement: Additional file 7 — The three populations, CD3+, CD4+ and CD8+ T cells, shared largely conserved expression patterns for the significant genes, demonstrated by the hierarchical clustering (using the Euclidian distance metric) of the combined 4167 significant genes upon T-cell activation in CD3+, CD4+ and CD8+ T-cell populations (average of three biological-replicate experiments for each population). Color denotes degree of differential expression comparing to 0 hour (saturated red = 3-fold upregulation, saturated green = 3-fold down-regulation, black = unchanged, gray = no data available). [file 1471-2164-9-225-S7.pdf]

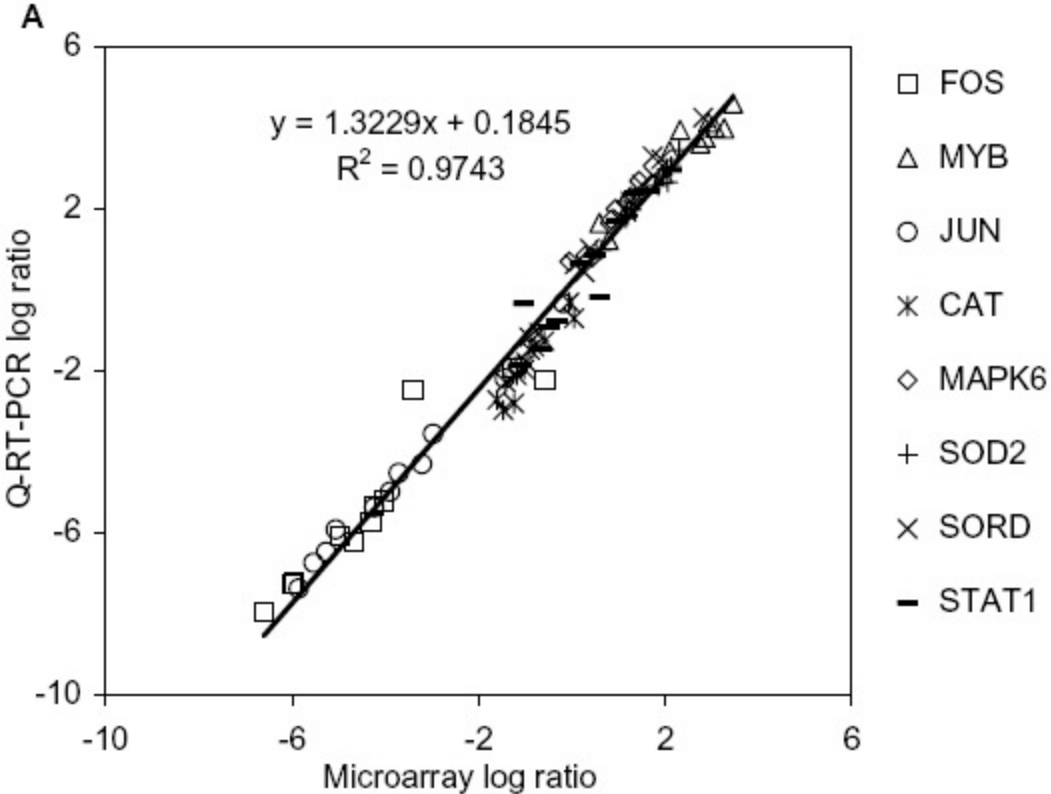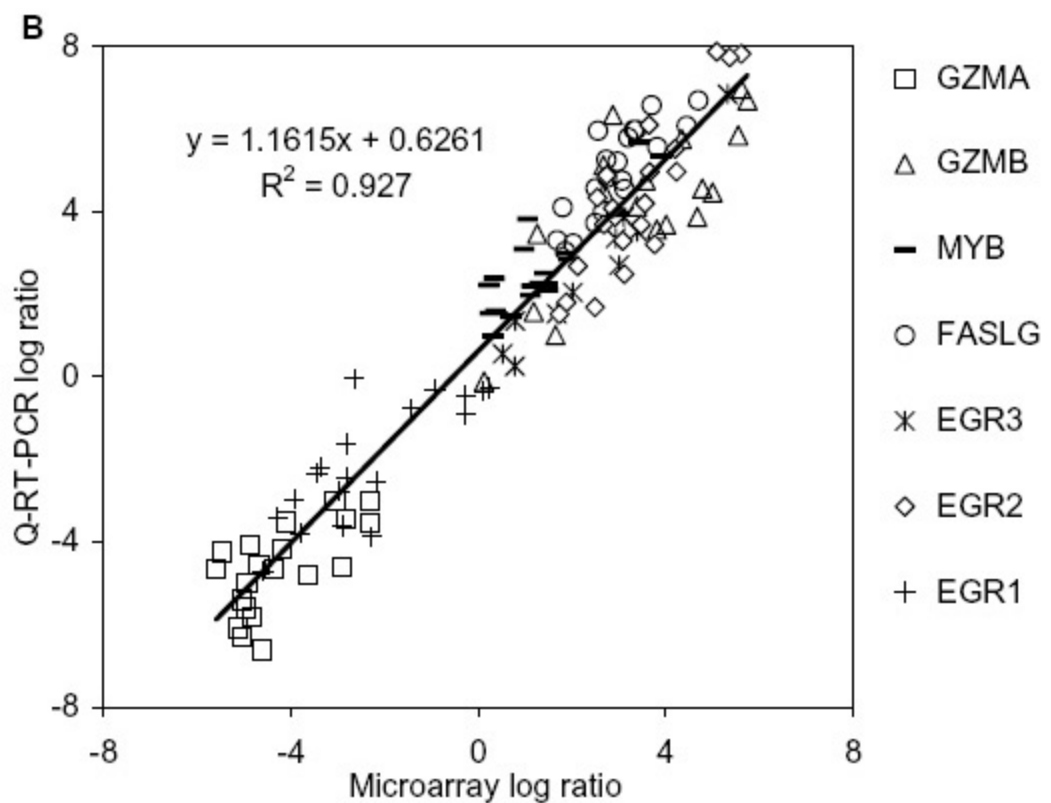

Supplement: Additional file 9 — Q-RT-PCR validation of microarray results across multiple culture samples. (A) Q-RT-PCR versus microarray log expression ratios (timepoint vs. 0 hour) from CD3+ T-cell activation experiments, E1–E3, (for all 12 (= 3 × 4) timepoints: 4, 10, 48 and 96 hours of 3 experiments) for each of the 8 selected genes (FOS, MYB, JUN, CAT, MAPK6, SORD, SOD2, and STAT1). (B) Q-RT-PCR versus microarray log expression ratios (timepoint vs. 0 hour) from CD4+ and CD8+ T-cell activation experiments, E8 and E9, (for all 20 (= 2 × 2 × 5) timepoints: 6, 12, 24, 48 and 72 hours of 2 experiments) for each of the 7 selected genes (EGR1, EGR2, EGR3, FASL, GZMA, GZMB, and MYB). [file 1471-2164-9-225-S9.pdf]
